# Long Non-coding RNA GAS5 Worsens Coronary Atherosclerosis Through MicroRNA-194-3p/TXNIP Axis

**DOI:** 10.1007/s12035-021-02332-x

**Published:** 2021-02-27

**Authors:** Yanbing Li, Yu Geng, Boda Zhou, Xuejiao Wu, Ou Zhang, Xiaonan Guan, Yajun Xue, Siyuan Li, Xianjing Zhuang, Jie Zhou, Meng Chang, Guobin Miao, Lizhong Wang

**Affiliations:** 1 Department of Cardiology, Beijing Chaoyang Hospital, Capital Medical University, Beijing, 100043 China; 2 Department of Cardiology, Beijing Tsinghua Changgung Hospital, Tsinghua University, No. 168 Litang Road, Changping District, Beijing, 102218 China; 3 School of Clinical Medicine, Tsinghua University, Beijing, China; 4 Cardiac Rehabilitation Center, Beijing Rehabilitation Hospital, Capital Medical University, Xixiazhuang, Badachu Road, Shijingshan Distract, Beijing, 100144 China

**Keywords:** Coronary atherosclerosis, Long non-coding RNA growth arrest-specific 5, MicroRNA-194-3p, Thioredoxin-interacting protein, Endothelial cells, Proliferation, Apoptosis

## Abstract

It is formerly conducted that long non-coding RNA growth arrest-specific 5 (GAS5) is involved in the process of coronary atherosclerosis (AS). The regulatory effects of GAS5 on the microRNA (miR)-194-3p/thioredoxin-interacting protein (TXNIP) axis in AS have been insufficiently explored yet. Thereafter, this work is started from GAS5/miR-194-3p/TXNIP axis in AS. AS rats were modeled to obtain their coronary vascular tissues and endothelial cells (ECs), in which GAS5, miR-194-3p, and TXNIP expression were tested. ECs were identified by immunohistochemistry. The mechanism among GAS5, miR-194-3p, and TXNIP was determined. ECs were transfected with inhibited GAS5 or overexpressed miR-194-3p to decipher their functions in proliferation and apoptosis of ECs in AS. Raised GAS5 and TXNIP and degraded miR-194-3p expression levels exhibited in AS. GAS5 bound to miR-194-3p while miR-194-3p targeted TXNIP. Depleting GAS5 or restoring miR-194-3p enhanced proliferation and depressed apoptosis of ECs in AS. This work clearly manifests that inhibited GAS5 facilitates the growth of ECs through miR-194-3p-targeted TXNIP in AS, consolidating the basal reference to the curing for AS.

## Introduction

Atherosclerosis (AS) is defined as a chronic inflammatory disease within the arterial wall, consequently resulting in peripheral artery disease, myocardial infarction, and coronary artery disease [1]. Pathologically, AS is mainly evoked from dyslipidemia, insulin resistance, hyperglycemia, oxidative stress, and inflammation [2]. Actually, various cells including endothelial cells (ECs) and smooth muscle cells are substantially implied to connect with the generation of atherosclerotic plaques [3]. Specifically, the dysfunction of ECs comprises of non-adaptive changes in functional phenotypes, which are indicative in regulating thrombosis, hemostasis, local vascular tone and redox balance, and internal acute and chronic inflammatory responses [4]. Thereby, targeting ECs to improve their functions is potentially essential to manage AS.

It is instructive that long non-coding RNA growth arrest-specific 5 (GAS5) plays a role in inflammatory diseases, apoptosis of vascular ECs, and AS [5]. Besides that, GAS5 has been implied in the progression of AS through acting in the field of cholesterol reverse-transport and intracellular lipid accumulation [6]. Also, it is specifically elucidated that GAS5 influences autophagy dysfunction of ECs in AS [7]. Moreover, Shen S et al. have found that GAS5 performs crucially in the process of lipid metabolism and inflammation in AS via modulating microRNA (miR)-135a [8], indicating the co-expression of lncRNAs and miRNAs in AS. MiRNAs can alter atherosclerotic plaque progression/regression balance through the modification of signaling and lipid homeostasis pathways [9]. Pertaining to the group of miRNAs, miR-194 is reported to restrict inflammatory response and impair the permeability of human dermal microvascular ECs [10]. Functionally, miR-194 is involved in the transition of astrocytes into ECs and participates in gene regulation of AS [11]. Thioredoxin-interacting protein (TXNIP), an endogenous inhibitor of thioredoxin, is documented to regulate diabetes-accelerated AS [12]. Furthermore, knocking down TXNIP functions to reduce the atherosclerotic lesion, implying its therapeutic potential in AS and inflammatory diseases [13]. Serving as the target gene of miR-20a, TXNIP can mediate inflammation in human aortic ECs, thereby to prevent the development of AS [14]. Concerning to that, it is a significant issue needed to be solved that whether GAS5 may exert as the regulator of miR-194-3p, which further targets the potential downstream gene TXNIP to affect AS. Thus, this work was initiated to explain how GAS5/miR-194-3p/TXNIP axis functioned in the progression of AS.

## Methods and Materials

### Ethics Statement

All animal experiments were executed with reference to the Guidelines for the Care and Use of Laboratory Animals (National Academy of Sciences Press, revised in 2010). The experimental operation was endorsed by the ethics committee of Beijing Tsinghua Changgung Hospital (ethical number: 201920629).

### Experimental Animals

Male Sprague Dawley rats (6 weeks old) of specific pathogen-free grade (Beijing Vital River Laboratory Animal Technology Co., Ltd., Beijing, China) were adaptively reared for 1 week (with sufficient food and water, 20-25°C, and relative humidity of 45-70%).

### AS Rat Modeling and Treatment

AS rats were raised for a week and injected with vitamin D_3_ (GenePharma, Shanghai, China), with a total dose of 70,000 U/kg for 3 days. Then, the rats were fed a high-fat diet containing 4% cholesterol, 5% glucose, 10% lard, and 82% normal diet for 21 days. The rats in the control group were fed a normal diet and intraperitoneally injected with physiological saline once a month. The biochemical changes of blood lipid were detected by an automatic biochemical analyzer after 12 weeks [15]. Rats were grouped as follows: control group (rats fed with standard food and drinking water), AS group (AS rats fed with high-fat diet and intraperitoneally injected with vitamin D_3_), sh-negative control (NC) group (AS rats fed with high-fat diet and intraperitoneally injected with vitamin D_3_, and injected with sh-GAS5 NC), sh-GAS5 group (AS rats fed with high-fat diet and intraperitoneally injected vitamin D_3_, and injected with sh-GAS5), agomir NC group (AS rats fed with high-fat diet and intraperitoneally injected vitamin D_3_, and injected with miR-194-3p agomir NC), and miR-194-3p agomir group (AS rats fed with high-fat diet and intraperitoneally injected with vitamin D_3_, and injected with miR-194-3p agomir). The transfection plasmids (10 μL, all from Huibaibio, Shenyang, China) were injected into rats (*n* = 12/group) through the caudal vein at 21 days post modeling, and the needle was kept in the vein for 1 min. Since that, the rats were continuously fed a high-fat diet [15].

### Specimen Collection

At 16 weeks post injection, all rats were euthanized to obtain coronary tissues. ECs were collected as previously described [16]. Coronary tissues were detached in 0.1% collagenase to isolate ECs and centrifuged at 1200 rpm to remove the supernatant. ECs were preserved at −80°C and identified by immunohistochemistry testing factor VIII-related antigen von Willebrand factor (vWF) [15].

### Hematoxylin-Eosin Staining

Coronary tissues were fixed in 10% formalin (NanJing JianCheng Bioengineering Institute, Nanjing, China) for 24 h, followed by dehydration with ethanol (Sigma-Aldrich, St. Louis, Missouri, USA), clearance with xylene, dewaxing, and embedment in paraffin. After that, the paraffin-embedded block was dewaxed and stained with hematoxylin staining solution. Next, the tissues were differentiated by 1% acidic alcohol, immersed in eosin solution, and dehydrated in ethanol. After clearance with xylene twice (10 min), the tissues were covered with *Acacia senegal* and observed under a CX-31 optical microscope (Olympus, Tokyo, Japan). An LM1235 ultra-thin semi-automatic slicer (Leica, Wetzlar, Germany) was utilized for sectioning while a KD-BM machine for tissue embedment (Kedee, Zhejiang, China).

### Transferase-Mediated Deoxyuridine Triphosphate-Biotin Nick End Labeling Staining

Transferase-mediated deoxyuridine triphosphate-biotin nick end labeling (TUNEL) staining was conducted with an in situ apoptosis detection kit (11684795910, Basel Roche, Switzerland). Paraffin-embedded sections were dewaxed and dehydrated, and treated with 20 mg/mL proteinase K and 3% H_2_O_2_ for 30 min. Then, the sections were soaked in 1% citric acid solution (0.1% Triton X-100) and supplemented with 50 mL TUNEL reaction mixture. The sections were treated with horseradish peroxidase-labeled goat anti-rabbit antibody and stained with diaminobenzidine (DAB). A color image analyzer (BI-2000) and a high-power microscope were applied for image analysis. In 5 randomly selected fields, the number of apoptotic cells was counted [17].

### Immunohistochemistry

Coronary vascular tissues were fixed, sectioned, and sealed. After that, tissues were reacted with the primary antibody vascular endothelial growth factor (VEGF, 1:100, sc-7269, Santa Cruz Biotechnology, Santa Cruz, CA, USA) and with goat anti-rabbit secondary antibody immunoglobulin G (IgG, ab150077, 1:200, Abcam, MA, USA) in phosphate-buffered saline (PBS). Then, the tissues were kept in streptavidin biotin-peroxidase complex (Boster Biological Technology Co. Ltd., Hubei, China), stained with DAB (Boster), and observed by a microscope (Leica) [18].

Immunohistochemistry was applied to determine vWF in ECs. The collected cells were placed in 4% paraformaldehyde, frozen in 30% sucrose PBS, and sectioned into 30 μm by a cryostat. Subsequently, cells were reacted with 0.3% H_2_O_2_ or methanol and sealed with 10% PBS. With En Vision two-step method, cells were probed with rabbit anti-human factor VII-related antigen polyclonal antibody (Neomarkers, CA, USA) and with anti-rabbit/mouse universal immunohistochemical kit (DAKO, MI, USA). Followed by that, cells were developed by DAB, counterstained by hematoxylin solution, treated with ammonia, and observed under an optical microscope (Olympus).

### Cell Culture and Transfection

ECs from rats in the control group and AS group were cultured in Dulbecco’s modified Eagle’s medium//high glucose (SH30022.01, Gibco, CA, USA) supplemented with 10% fetal bovine serum (HyCLone, UT, USA). Detached by trypsin (825M042, 1:250, Solarbio, China), ECs were passaged. Through restriction enzyme digestion and sequencing, miR-194-3p agomir, miR-194-3p agomir NC, sh-NC, or sh-GAS5 were synthesized and packaged in liposomes. ECs at passage 3 with 80% confluence were transfected with the diluted liposomes and cultured for 24 h [16]. The cell groups are as follows: control group (ECs from rats in the control group), AS group (ECs from rats in the AS group), sh-NC group (ECs from rats in the AS group were transfected with sh-GAS5 NC), sh-GAS5 group (ECs from rats in the AS group were transfected with sh-GAS5), agomir NC group (ECs from rats in the AS group were transfected with miR-194-3p agomir NC), and miR-194-3p agomir group (ECs from rats in the AS group were transfected with miR-194-3p agomir).

### Reverse Transcription Quantitative Polymerase Chain Reaction

Total RNA was extracted from coronary vascular tissues and ECs via Trizol reagent (ThermoFisher, Waltham, MA, USA) and then processed with reverse transcription via a cDNA transcription kit (ThermoFisher). U6 served as the internal control for GAS5 while glyceraldehyde-3-phosphate dehydrogenase served as the internal control for miR-194-3p and TXNIP. A SYBR-Green RT-PCR kit (Takara, Tokyo, Japan) was applied to test gene expression levels, which were further calculated by 2^−ΔΔCt^ method [19, 20]. Primers were listed in Table 1.

**Table 1:** Primer sequences

Primers	Forward (5′-3′)	Reverse (5′-3′)
GAS5	AGCTGGAAGTTGAAATG	CAAGCCGACTCTCCATA
miR-194-3p	CTCGCTTCGGCAGCACA	AACGCTTCACGAATTTGCGT
TXNIP	ACTCCTCAAGATGGGTGGCAATC	ACATCCACCCAGCAAACACTCCT
U6	CTCGCTTCGGCAGCACA	AACGCTTCACGAATTTGCGT
GAPDH	TGTGATGGGTGTGAACCACGAGAA	GAGCCCTTCCACAATGCCAAAGTT

*GAS5* long non-coding RNA growth arrest-specific 5; *miR-194-3p* microRNA-194-3p; *TXNIP* thioredoxin-interacting protein; *GAPDH* glyceraldehyde-3-phosphate dehydrogenase

### Western Blot Assay

Total protein was extracted from tissues and cells with radio-immunoprecipitation assay buffer, of which the protein concentration was measured by a bicinchoninic acid protein kit (ThermoFisher Scientific). Separated by 10% sodium dodecyl sulfate polyacrylamide gel electrophoresis, the protein was placed into a 0.45-μM polyvinylidene fluoride membrane (Millipore, Billerica, MA, USA) and probed with the specific antibodies. Protein bands were developed by enhanced chemiluminescence and photographed by an Image Quant LAS 4000C gel imager (General Electric Company, Boston, MA, USA). Primary antibodies are as follows: TXNIP (1:1000, ab188865, Abcam), VEGF (1:200, sc-7269, Santa Cruz Biotechnology), and vWF (1:1000, A0082, DAKO). Secondary antibody is anti-rabbit IgG (7074, 1:2000, Cell Signaling Technology) [21]. Glyceraldehyde-3-phosphate dehydrogenase (1:2500, ab9485, Abcam) was used as the internal control.

### Cell Counting Kit-8 Assay

ECs were cultured under standard conditions to 70% confluence and seeded in 96-well plates at 1 × 10^4^ cells/well. Cell proliferation was evaluated by a cell counting kit (CCK)-8 kit (Sigma, Tokyo, Japan). Optical density values were read at 450 nm [17].

### RNA Immunoprecipitation Assay

A Magna RNA immunoprecipitation (RIP) kit (Millipore Corporation, MA, USA) was used in RIP assay by following the protocols. ECs with 85% confluence were lysed in complete RIP lysis buffer and incubated with anti-AGO2 (ab32381) or rabbit IgG (ab172730, both from Abcam). The purified RNA was detected by reverse transcription quantitative polymerase chain reaction (RT-qPCR) [22].

### Dual Luciferase Reporter Gene Assay

The binding site of miR-194-3p with GAS5 or TXNIP was predicted by Jefferson website (https://cm.jefferson.edu/rna22/Precomputed/). The 3′-untranslated region (UTR) sequences of GAS5 and TXNIP were synthesized, followed by digestion, purification, ligation with pGL3-control vector, transformation, and sequencing. GAS5-wild type (WT) 3′-UTR and TXNIP-WT 3′-UTR were mutated to produce GAS5-mutant (MUT) 3′-UTR and TXNIP-MUT 3′-UTR. Following the instructions of X-tremegeneHP Transfection Reagent (Roche), GAS5-WT/GAS5-MUT, TXNIP-WT/TXNIP-MUT, and miR-194-3p-agomir/agomir-NC were added into opti-minimum essential medium and transfected into ECs. After 48 h of culture, the luciferase activity was measured by dual luciferase reporter gene assay (Promega, Madison, WI, USA). The relative activity of firefly luciferase was normalized to Renilla luciferase [16].

### Flow Cytometry

ECs at logarithmic growth were detached upon 80% confluence and seeded into 6-well plates at 5 × 10^4^ cells/well. At 72 h post cultivation, trypsinized ECs were centrifuged at 1000 r/min and resuspended in pre-chilled PBS. ECs were centrifuged once again and suspended in 5 mL binding buffer, followed by staining with 5 μL annexin V-fluorescein isothiocyanate and propidium iodide (PI) solution. ECs were then detected by a flow cytometer (BD Biosciences, Franklin Lakes, USA) to calculate the apoptosis rate.

When determining cell cycle distribution, ECs were pre-treated as described above. Then, the detached ECs were fixed with 70% ethanol and incubated with 20 mg/L RNaseA (prepared by PBS with 0.2% Triton X-100) and with 50 mg/L PI. Cell cycle distribution was analyzed by the ModFit LT software (Becton, Dickinson and Company, NJ, USA).

### Statistical Analysis

All data were statistically analyzed by the SPSS21.0 (IBM, NY, USA) statistical software. The measurement data were expressed as the mean ± standard deviation. Independent sample *t* test was applied for comparing the discrepancy between two groups. With *P* < 0.05, statistical significance was established.

## Results

### Increased vWF and VEGF, Inhibited Proliferation of ECs, and Promoted Apoptosis Are Found in AS

AS-modeled rats were established by vitamin D3 injection and high-fat diet. Then, coronary vascular tissues and ECs were collected for subsequent experiments.

**Fig. 1:**
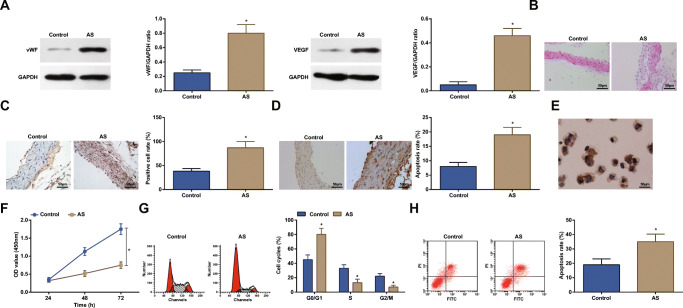
Increased vWF and VEGF, inhibited proliferation of ECs, and promoted apoptosis are present in AS. **a** Western blot assay detected vWF and VEGF protein expression in ECs. **b** H&E staining observed the morphology of ECs in coronary vascular tissues. **c** Immunohistochemistry detected VEGF expression in ECs. **d** TUNEL staining detected the apoptosis of ECs. **e** Immunohistochemistry identified ECs. **f** CCK-8 assay detected the proliferation of ECs. **g** and **h** Flow cytometry detected cell cycle distribution and apoptosis of ECs. * *P* < 0.05 compared with the control group; the measurement data were expressed as the mean ± standard deviation. Paired *t* test was applied for discrepancy between the two groups. *N* = 3

Related studies have evidenced that high expression of vWF and VEGF deteriorates AS, and vWF participates in the formation of thrombosis [23, 24]. Concerning to that, vWF and VEGF protein expression in coronary vascular tissues were measured, and the results found that they were both upregulated in AS (Fig. 1a). Hematoxylin-eosin (H&E) staining, immunohistochemistry, and TUNEL staining (Fig. 1b-d) detected that endothelial tissue structure was disorder, atherosclerotic plaques and thrombosis were obvious, and VEGF positive rate and apoptosis rate of ECs were highlighted in AS rats.

Based on the fact that vWF is a glycoprotein specifically secreted by ECs, which is stored in the Weibel-Palade body and possessed the capacity to identify ECs [25], immunohistochemistry was applied to identify vWF (Fig. 1e). It was demonstrated that the collected cells showed brownish-yellow cytoplasm, dense nucleus, and clear cell outline. Thus, those cells were confirmed as ECs. CCK-8 assay and flow cytometry (Fig. 1f-h) depicted that impaired proliferation of ECs, increased G0/G1 cell phase, shortened S and G_2_/M cell phases, and enhanced cell apoptosis were seen in AS rats. Those findings proved that AS was successfully modeled in rats.

### Depleting GAS5 Enhances Proliferation and Depresses Apoptosis of ECs in AS

**Fig. 2:**
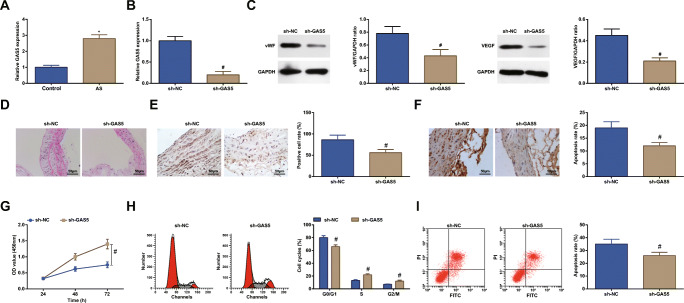
Depleting GAS5 enhances proliferation and depresses apoptosis of ECs in AS. **a** and **b** RT-qPCR detected GAS5 expression in ECs. **c** Western blot assay detected vWF and VEGF protein expression in ECs. **d** H&E staining observed the morphology of ECs in coronary vascular tissues. **e** Immunohistochemistry detected VEGF expression in ECs. **f** TUNEL staining detected the apoptosis of ECs. **g** CCK-8 assay detected the proliferation of ECs. **h** and **i** Flow cytometry detected cell cycle distribution and apoptosis of ECs. * *P* < 0.05 compared with the control group; # *P* < 0.05 compared with the sh-NC group; the measurement data were expressed as the mean ± standard deviation. Paired *t* test was applied for discrepancy between the two groups. *N* = 3

The existing literature has indicated that GAS5 knockout prevents the progression of AS [6]. Detected by RT-qPCR, it was displayed that GAS5 was overexpressed in ECs from AS rats (Fig. 2a).

GAS5 intervention assay was conducted to explore the impacts of GAS5 on coronary vascular tissues and ECs. RT-qPCR presented that (Fig. 2b) GAS5 interference plasmid reduced GAS5 expression in ECs, suggesting successful downregulation of GAS5. As a result of GAS5 downregulation in ECs, vWF and VEGF protein expression were inhibited, the arrangement of ECs was improved, VEGF positive rate and apoptosis rate were decreased, proliferation of ECs was increased, cells in G0/G1 phase were reduced, and cells in S and G_2_/M phases were increased (Fig. 2c-i).

### GAS5 Targets miR-194-3p

**Fig. 3:**
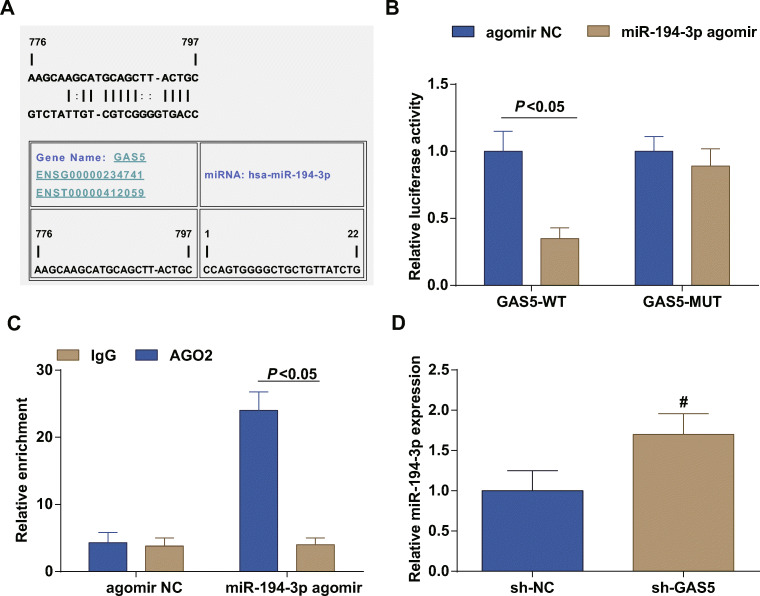
GAS5 targets miR-194-3p. **a** Jefferson website predicted the binding site between GAS5 and miR-194-3p. **b** Dual luciferase reporter gene assay verified the targeting relation between GAS5 and miR-194-3p. **c** RIP assay tested the relative enrichment of GAS5 and miR-194-3p. **d** RT-qPCR detected miR-194-3p expression after GAS5 inhibition. # *P* < 0.05 compared with the sh-NC group; the measurement data were expressed as the mean ± standard deviation. Paired *t* test was applied for discrepancy between the two groups. *N* = 3

miR-194-3p has been implicated to involve in the occurrence of coronary heart disease [26]. The regulatory mechanism between GAS5 and miR-194-3p was firstly decoded by predicting their binding site through Jefferson website (Fig. 3a), and subsequently validated by dual luciferase reporter gene assay (Fig. 3b). GAS5-WT/miR-194-3p-agomir transfection diminished the luciferase activity of cells while GAS5-MUT/miR-194-3p-agomir transfection caused no difference in the luciferase activity. Anti-AGO2 RIP was performed in ECs which had transfected with miR-194-3p-agomir or agomir-NC (Fig. 3c). GAS5 and miR-194-3p were enriched in the AGO2. As manifested by RT-qPCR (Fig. 3d), miR-194-3p expression was elevated after GAS5 inhibition.

### Restoring miR-194-3p Reinforces Proliferation and Impedes Apoptosis of ECs in AS

**Fig. 4:**
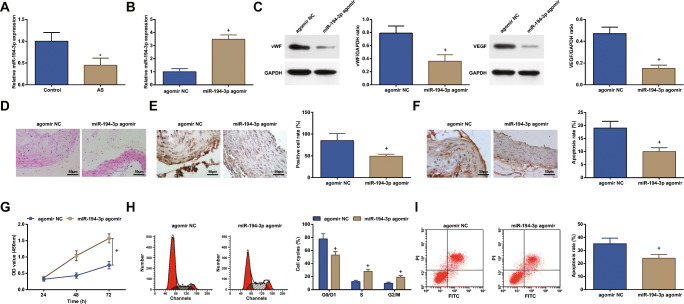
Restoring miR-194-3p reinforces proliferation and impedes apoptosis of ECs in AS. **a** and **b** RT-qPCR detected miR-194-3p expression in ECs. **c** Western blot assay detected vWF and VEGF protein expression in coronary vascular tissues. **d** H&E staining observed the morphology of ECs in coronary vascular tissues. **e** Immunohistochemistry detected VEGF expression in ECs. **f** TUNEL staining detected apoptosis of ECs. **g** CCK-8 assay detected proliferation of ECs. **h** and **i** Flow cytometry detected cell cycle distribution and apoptosis of ECs. * *P* < 0.05 compared with the control group; + *P* < 0.05 compared with the agomir NC group; the measurement data were expressed as the mean ± standard deviation. Paired *t* test was applied for discrepancy between the two groups. *N* = 3

The involvement of miR-194-3p in the growth of ECs of coronary vascular tissues was disclosed. As detected, miR-194-3p was lowly expressed in ECs in AS (Fig. 4a, b). In this study, transfection of miR-194-3p agomir elevated miR-194-3p expression, hinting the successful upregulation of miR-194-3p in ECs.

Functionally, miR-194-3p agomir in ECs was found to decrease vWF and VEGF protein expression and VEGF positive rate, attenuate structural damage of coronary vascular tissues, suppress apoptosis rate, reinforce cell proliferation, reduce cells in the G0/G1 phase, and increase cells in the other two phases (Fig. 4c-i).

### GAS5 Regulates TXNIP Through miR-194-3p

**Fig. 5:**
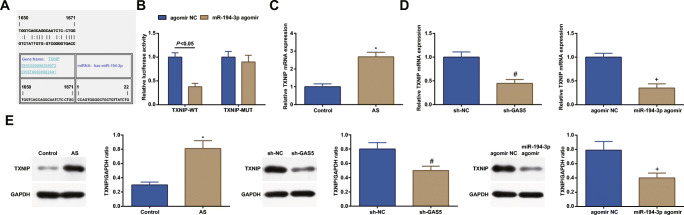
GAS5 regulates TXNIP through miR-194-3p. **a** Jefferson predicted the binding site between miR-194-3p and TXNIP. **b** Dual luciferase reporter gene assay verifies the targeting relation between miR-194-3p and TXNIP. **c** and **d** RT-qPCR detected TXNIP expression. **e** Western blot assay detected TXNIP protein expression. * *P* < 0.05 compared with the control group; # *P* < 0.05 compared with the sh-NC group; + *P* < 0.05 compared with the agomir NC group; the measurement data were expressed as the mean ± standard deviation. Paired *t* test was applied for discrepancy between the two groups. *N* = 3

A relevant research has pointed out that TXNIP expression is higher in AS while TXNIP knockout could attenuate AS [13]. To further investigate the regulatory mechanism downstream of miR-194-3p, Jefferson website predicted the binding site between miR-194-3p and TXNIP (Fig. 5a) while dual luciferase reporter gene assay validated the targeting relation between those two factors (Fig. 5b). Experimentally, miR-194-3p agomir destroyed the luciferase activity of TXNIP-WT in cells while it had no impact on that of TXNIP-MUT. Given that, it was speculated that miR-194-3p could directly inhibit TXNIP.

To clarify that whether GAS5 regulated TXNIP via miR-194-3p, RT-qPCR and western blot assay were implemented to test TXNIP expression in ECs (Fig. 5c). Clearly, TXNIP expression reached a high level in AS. Furthermore, TXNIP expression was downregulated after intervening GAS5 or upregulating miR-194-3p (Fig. 5d, e). The above results indicated that GAS5 regulated TXNIP via miR-194-3p.

## Discussion

**Fig. 6:**
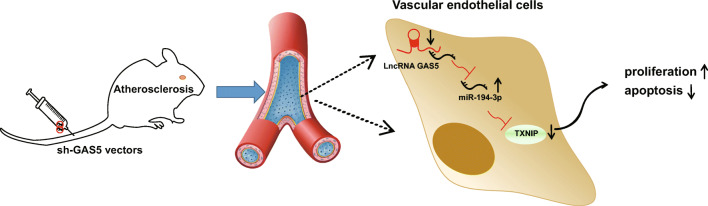
Schematic diagram. Interfering with GAS5 inhibits the adsorption of GAS5 on miR-194-3p. Upregulating miR-194-3p regulates TXNIP expression, thereby protecting ECs, and reducing the formation of atherosclerotic plaques in AS

AS is a common cardiovascular disease, predominantly contributing to heart attack and stroke [27]. Studies have more or less mentioned the independent role of GAS5, miR-194-3p, and TXNIP in this disease, but limited to their combined reciprocal. This study has recognized the effects of GAS5/miR-194-3p/TXNIP axis in AS and extracted the summary that depleted GAS5 upregulated miR-194-3p to inhibit TXNIP, thereby to promote growth of ECs and reduce the formation of atherosclerotic plaques in AS (Fig. 6).

Firstly, we tested GAS5 expression and found that it was upregulated in ECs of AS rats. Next, to decode the mechanism of GAS5 in the growth of ECs, ECs were interfered with GAS5, and then it was found that vWF and VEGF contents were reduced, apoptosis was restrained, G0/G1 cells increased, and proliferation ability of ECs was strengthened. Commonly, studies have evidenced that GAS5 is highly expressed in AS patients and modeled rats [28, 29]. Explored by a late work, GAS5 expression is manifested with an increment in AS, which promotes the progression of AS in vivo [6]. Supplemental to the finding, another research has pictured that raised GAS5 expression is obviously demonstrated in AS-modeled mice and ablating GAS5 disturbs the progression of AS [8]. In addition to that, AS patients and oxidized low-density lipoprotein-treated human aortic ECs both express GAS5 at a high level, and GAS5 depletion disrupts the apoptosis of human aortic ECs [7]. Also, a creative work has reported the elevated GAS5 expression in atherosclerotic plaques, and silencing GAS5 would interfere the apoptosis of ECs via exosomes [30].

To proceed, the molecular regulation of GAS5 on its binding genes revealed that GAS5 can specifically target miR-194-3p. Then, to elucidate the concrete functions of miR-194-3p in AS, miR-194-3p upregulation was projected on ECs to discover that restored miR-194-3p functioned as similar as depleted GAS5 in ECs in AS. At present, nearly no research has recorded the binding relation between GAS5 and miR-194-3p. It has been elaborated that miR-194 ablation is negatively connected with the growth of monocyte cells THP-1 in an intracellular inflammatory model [26]. Echoed with the present work, another work has documented that miR-194-3p expression is lower in cigarette-inflamed bronchial epithelium, and overexpressing miR-194-3p hinders apoptosis of human bronchial ECs [31]. Similarly, the reduction is noticeable in miR-194-1 expression in rats having experienced stroke [32].

Subsequently, the downstream regulatory mechanism of miR-194-3p was predicted and verified. The results manifested that miR-194-3p targeted and mediated TXNIP. Moreover, the upregulated TXNIP in AS was repressed by miR-194-3p upregulation or GAS5 downregulation, hinting that GAS5 mediated TXNIP through miR-194-3p. There is a lack of sufficient researches to identify the targeting relation between miR-194-3p and TXNIP, which needs more experiments for confirmation. TXNIP displays an increment in its expression in AS [33]. In compatibility with the finding, elevated TXNIP2 mRNA expression is characterized in acute myocardial infarction patients versus to the normal controls [34]. Besides, TXNIP expression is heightened in AS and silencing it diminishes vascular smooth muscle cell inflammation and attenuates AS [13]. Concretely, a documented report has stressed out that TXNIP expression is elevated in ECs of human carotid plaques, resulting in cell senescence [35].

## Conclusion

To sum up, this research has worked out that GAS5 ablation or miR-194-3p restoration restrains TXNIP to enhance the growth of ECs in AS, exerting a referential base for developing therapeutic agents for AS. For confirmation and further outspread of the results summarized in this work, much researches are required to conduct in a scientific and logical manner.

## Data Availability

Not applicable.
